# Enrichment of Viral Nucleic Acids by Solution Hybrid Selection with Genus Specific Oligonucleotides

**DOI:** 10.1038/s41598-017-10342-w

**Published:** 2017-08-29

**Authors:** Andrei A. Deviatkin, Alexander N. Lukashev, Mikhail M. Markelov, Larisa V. Gmyl, German A. Shipulin

**Affiliations:** 1 Chumakov Federal Scientific Center for Research and Development of Immune and Biological Products of Russian Academy of Sciences, Moscow, Russian Federation; 2 Research Institute of Occupational Health, Moscow, Russian Federation; 3 Institute of Molecular Medicine, Sechenov First Moscow State Medical University, Moscow, Russia; 4 Federal Budget Institute of Science Central Research Institute for Epidemiology, Moscow, Russian Federation

## Abstract

Despite recent advances, our knowledge of potential and rare human pathogens is far from exhaustive. Current molecular diagnostic tools mainly rely on the specific amplification of marker sequences and may overlook infections caused by unknown and rare pathogens. Using high-throughput sequencing (HTS) can solve this problem; but, due to the extremely low fraction of pathogen genetic material in clinical samples, its application is only cost-effective in special, rather than routine, cases. In this study, we present a method for the semi-specific enrichment of viral conservative sequences in a HTS library by hybridization in solution with genus-specific degenerate biotinylated oligonucleotides. Nucleic acids of the test viruses (yellow fever virus and Japanese encephalitis virus) were enriched by solution hybrid selection using pan-flavivirus oligonucleotides. Moreover, enterovirus (family: *Picornaviridae*, genus: *Enterovirus*) sequences were successfully enriched using foot-and-mouth disease virus (family: *Picornaviridae*, genus: *Aphthovirus*) oligonucleotide. The enrichment factor relative to the background nucleic acid was about 1,000-fold. As hybridization has less stringent oligonucleotide match requirements than PCR, few oligonucleotides are sufficient to cover the potential sequence variation in the whole genus and may even enrich nucleic acids of viruses of other related genera. Efficient enrichment of viral sequences makes its use in diagnostics cost-efficient.

## Introduction

Increasing human-animal interactions, migration and rising population density have resulted in the growing threat from emerging infections. Novel pathogens are among the major challenges to public health^1^.

The majority of emerging infectious diseases (EIDs) among humans are viral zoonoses. This is exemplified by recent outbreaks of zoonotic diseases with high lethality rates (SARS and MERS coronaviruses, ebolavirus etc.)^2^. Fast and accurate identification of infectious agents is a critical step in any surveillance strategy.

Most of the current diagnostic systems, such as PCR, NASBA and LAMP, are based on the specific amplification of nucleic acids (NAs)^3, 4^. However, all these methods have one serious limitation: diagnostic systems efficiently detect specific NA markers only if the pathogen genome was previously identified, sequenced and annotated^5^.

According to some estimates, there are hundreds of thousands viruses awaiting discovery^6^. Some of these viruses can pose a real threat to human health. HTS techniques (also termed next-generation sequencing, NGS) can directly identify genetic markers of unknown infectious agents in a pool of NAs from biological samples without using classical time-consuming virological or microbiological methods. However, total NA sequencing is not used in routine infectious disease diagnostics because of certain limitations. The genetic markers of a pathogen usually account for only a few reads among a huge number of background host-related sequences (human/animal and non-pathogenic environmental bacteria)^7^. For example, when HTS was used to identify the Schmallenberg virus, only192 out of 94,722 reads (0.2%) were mapped to its genome^8^.

The following strategies have been developed to increase the viral/host NA ratio before or after NA extraction: physical enrichment methods (ultrafiltration, centrifugation, virus particles precipitation); biological methods (exonuclease treatment); host NA depletion^7, 9–12^. Some tools for viral NA enrichment are based on PCR with non-specific or genus-specific degenerate oligonucleotide primers. The preferential amplification of pathogenic sequence (PATHseq) is based on using a set of 88 specific 8-mer oligonucleotides (which do not match the sequences of the 2,000 most abundant human transcripts) as primers for the synthesis of secondary cDNA strands^13^. Alternatively, the VIDISCA method based on the presence of virus-specific endonuclease restriction sites was suggested^14^. Advanced VIDISCA uses non-rRNA binding hexamers in a reverse transcription (RT) reaction^15^. It is also possible to enrich viral sequences by a broad-range RT-PCR using a set of degenerate oligonucleotides^16, 17^.

The recently developed virome capture sequencing platform for vertebrate viruses (VirCapSeq-VERT) technique increases the sensitivity of sequence-based virus detection by HTS^18^. The authors described target selection of genome sequences of known viruses by hybridization in a solution using 1,993,200 biotinylated oligonucleotides representing 342,438 viral coding sequences. However, as this approach used specific (non-degenerate) oligonucleotides, theoretically, it can only detect viruses that have regions with > 80% sequence identity to known viruses.

In this study, we describe a cost-effective method for the significant enrichment of conservative fragments of viral genomes by hybridization with degenerate genus-specific biotinylated oligonucleotides. We analyze the utility of the technique for NA library enrichment in order to increase HTS efficiency and decrease the cost per sample.

## Materials and Methods

### Selection of probe sequences

All full-genome flavivirus sequences available in Genbank in October 2014 were extracted. After alignment with MAFFT^19^, sequences sharing more than 90% identity were discarded. The data set was refined manually to exclude pegiviruses and incomplete sequences. Foot-and-mouth disease virus alignment was prepared similarly. Hybridization oligonucleotides matching the most conserved genome fragments were designed according to the following principles:

-length between 50–90 nt

- 90% match with all target sequences (degenerate nucleotides were used where necessary)

- whenever possible, degenerate positions were simplified to G or K (T/G), given that mismatches involving T and G result in a minimal penalty to hybridization efficiency^20^.

The potential ability of oligonucleotides to hybridize with selected target sequences was checked using the Mfold web server^21^.

Finally, three pan-flavivirus biotinylated oligonucleotides were chosen: Flavi-8469 ATGACNGAYACYACNVCNTTTGGRCARCARMGRGTKTTYAARGARAARGTKGACAC (genome positions: 8,709–8,764 in KF297915); Flavi-8844 GCNAARGGNAGNMGNGCYATNTGGTWYATGTGGCTNGGNDSNMGNTTYYTNGARTTYGARGCYYTNGGNTTYYTNAAYGARGAYCAYTGG (9,084–9,173 in KF297915); and Flavi-9834 GGNGMNTGGATGACNACWGANGACATGYTNNNNGTNTGGAAYVRRGTNTGGAT (10,092–10144 in KF297915).

The hybridization probe aimed at all FMDV sequences was designed similarly: ggTggKWTgCCKTCTggKTgTKCTggKACYTSTgTgTTKAATTCAATKWTKAA (genome positions: 6,803–6,855 in X80059).

### Samples, nucleic acid extraction and preparation of artificial samples containing human RNA and viral RNA

Donor whole blood samples were collected with written informed consent from volunteers in the CRIE (Central Research Institute for Epidemiology, Moscow). All experiments were performed in accordance with relevant guidelines and regulations and were approved by the CRIE ethics committee. Total human RNA was extracted from the human mononuclear cell fraction of the samples using the RNeasy Mini Kit (Qiagen).

The Gagar Japanese encephalitis virus (JEV) strain was cultured in pig embryo kidney cells. The 17D yellow fever virus (YFV) strain was passaged in Vero cells. The Gregory Echovirus 11 (E11) strain, which was originally obtained from ATCC, was cultured in human rhabdomyosarcoma cells. Viral RNA was extracted from cell culture medium using the Viral RNA Mini Kit (Qiagen).

The relative concentrations of isolated viral RNA and host RNA were estimated using in-house qPCR assays. Then, total human RNA (100 ng) was mixed with either 20 pg of YFV, 30 pg of JEV or 100 pg of E11. The total volume of each sample was adjusted to 10 µL with water.

### Preparation of cDNA libraries

First-strand cDNA was synthesized from RNA using Reverta-L RT kit (AmpliSens) according to the manufacturer’s instructions. The kit uses MMLV reverse transcriptase and random hexamer primers.

Second-strand cDNA synthesis was performed by adding 10 µL of RT mixture, 8 µL of 5x buffer C (AmpliSens), 5 µL of dNTP (2.5 mM) (AmpliSens), 1 µL of random hexamer oligonucleotide (168 pmol/µL) (in-house) and 1 µL of RNAse H (New England Biolabs), made up to 50 µL with milli-Q water. The mixture was incubated at 37 °C for 10 min. The enzymes were inactivated by heating at 95 °C for 2 min. After cooling on wet ice, 1 µL of Bst polymerase (New England Biolabs) and 0.5 µL of Klenow exo- (New England Biolabs) were added. Incubation conditions were as follows: 4 °C for 45 secs, 20 °C for 5 min, 37 °C for 5 min, 45 °C for 5 min, 50 °C for 5 min and 68 °C for 5 min. The reaction product was purified with Agencourt AMPure XP beads (Beckman Coulter) using the manufacturer’s protocol, with the exception of an altered bead-to-DNA ratio, where we used a 1.5:1 beads-to-DNA solution ratio, instead of a 1.8:1 ratio. DNA was eluted to 17 µL of milli-Q water.

To incorporate sequencing adapters, purified cDNA libraries (15 µL) were mixed with 5 µL of 5x transposase buffer (in-house) and 0.5 µL of the transposase and transposon complex, termed transposome (in-house)^22^. Total reaction volume was adjusted with milli-Q water up to 25 µL. The mixture was incubated at 55 °C for 5 min. The reaction was stopped by adding 1 µL of 200 mM EDTA. The reaction product was purified with Agencourt AMPure XP beads (Beckman Coulter) using the manufacturer’s protocol. DNA was eluted to 22 µL of milli-Q water.

The cDNA libraries were amplified by real-time PCR. PCR reactions (50 µL) included 200 nM of NTS1 primer (5′ TCG TCG GCA GCG TCA GAT GTG TAT AAG AGA CAG 3′), 200 nM of NTS2 primer (5′ GTC TCG TGG GCT CGG A GAT GTG TAT AAG AGA CAG 3′), 0.25 mM of dNTP, 14 µL of PCR-mix-2-FL (AmpliSens), 1.2X of EvaGreen (Biotium Inc.), and a 20 µL purified cDNA sample.

The oligonucleotides (total volume: 5 µL) were separated from other parts of the PCR mixture (total volume: 45 µL) by a wax layer. Amplification conditions were as follows: 30 °C for 1 min; 37 °C for 1 min; 72 °C for 1 min; denaturation at 95 °C for 5 min; 18 cycles at 95 °C for 20 s, 65 °C for 30 s and 72 °C for 60 s. Real-time PCR amplification was performed using a Rotor Gene 6000 instrument (Qiagen). The amplification was monitored to stop the reaction during the exponential phase and before the reaction reached the plateau^23^.

Aliquots of PCR product were stored at −20 °C for further analysis.

### Solution hybrid selection of viral NAs

The cDNA libraries containing human and YFV or human and JEV genetic material were hybridized with three pan-flavivirus biotinylated oligonucleotides (Flavi-8469, Flavi-8844, Flavi-9834). The library containing human and E11 genetic material was hybridized with FMDV-specific biotinylated oligonucleotide.

Hybrid selection was performed similarly to a previously published method^24^ with minor modifications.

Briefly, a 20 µL mix containing 1X hybridization buffer (0.25 M sodium phosphate buffer: pH 7.0, 0.5% SDS, 5 mM EDTA, 1xSSC, 2xDenhardt’s solution), biotinylated oligonucleotide(s) (final concentration: 20 nM each), 1 µg Cot-1 DNA (Invitrogen), 50 uM oligonucleotide Nts1_SpC3 (5′ CTG TCT CTT ATA CAC ATC TGA CGC TGC CGA CGA spC3 3′), 50 uM oligonucleotide Nts2_SpC3 (5′ CTG TCT CTT ATA CAC ATC TCC GAG CCC ACG AGA C spC3 3′) and 3 µL of an amplified cDNA library was heated at 97 °C for 5 min. Next, the mixture was slowly cooled to 47 °C (3 °C every 10 min). After 15 h at 47 °C, the hybridization mixture was added to 100 µg of M-270 streptavidin Dynabeads (Invitrogen), which had been initially processed as follows: the beads were washed twice in 150 µL of a buffer containing 2 M NaCl, 10 mM Tris-HCl (pH 7.5) and 1 mM EDTA, then incubated in 14 µL of a solution containing 1X hybridization buffer, 2.2% casein (Stereospecific Detection Technologies GmbH) and 100 mg/L of Cot-1 DNA (Invitrogen) for 60 min at 37 °C, and then resuspended in 80 µL of a buffer containing 0.4 M NaCl and 6.25 mM Tris-HCl (pH 7.5).

After 15 min at 37 °C, the beads were pulled down by the magnetic rack and washed once at 47 °C for 5 min with 1 mL of prewarmed 1xSSC (saline and sodium citrate)/0.1% SDS (sodium dodecyl sulfate). Next, the beads were washed twice at 47 °C for 5 min with 1 mL of prewarmed 0.2xSSC/0.1%SDS, once at room temperature for 5 min with 1 mL 0.2xSSC/0.1%SDS, and once at room temperature for 5 min with 1 mL of 0.2xSSC/0.1% Tween 20. The beads were resuspended once after each washing step and collected by the magnetic rack. Hybrid-selected DNA, which was attached to the beads, was resuspended with 20 µL of 0.1% Tween 20.

The cDNA libraries captured on the beads were reamplified by PCR. PCR reactions (50 µL) consisted of 200 nM NTS1 primer (5′ TCG TCG GCA GCG TCA GAT GTG TAT AAG AGA CAG 3′), 200 nM NTS2 primer (5′ GTC TCG TGG GCT CGG A GAT GTG TAT AAG AGA CAG 3′), 0.25 mM dNTP, 14 µL of PCR-mix-2-FL (AmpliSens), 3 µL of 20X EvaGreen (Biotium Inc.) and 20 µL of bead suspension.

The oligonucleotides and dNTP (total volume: 13 µL) were separated from other components of the PCR mixture (total volume: 37 µL) by a wax layer. Amplification conditions were as follows: denaturation at 95 °C for 5 min; 19 cycles at 95 °C for 20 s, 65 °C for 40 s and 72 °C for 60 s. Real-time PCR amplification was performed using the Rotor Gene 6000 instrument (Qiagen).

### Enrichment evaluation by specific qPCR

The relative concentration of viral cDNA before and after hybridization was measured by a specific qPCR using GAPDH (Glyceraldehyde-3-Phosphate Dehydrogenase) as the housekeeping gene. The cycle threshold (Ct value) was chosen as being at the middle of the exponential phase of the amplification curve. The ratio between the host and viral NAs was assessed in terms of the Ct value difference. Oligonucleotide primers and probes for viral cDNA quantification (Table 1) were chosen in close proximity to the location of hybridization oligonucleotide probes. The efficiency of PCR amplification was assumed to be 100% (twofold amplification on each cycle) for further calculations.

**Table 1:** Primers and probes used for specific qPCR.

Primer name	Sequence 5′−3′	Position in the reference genome
YFV 8740F	AGCGGGAACTAGGAAGATCATGAAAGTT	8,740–8,767 in KF907504
YFV 8881R	TTCCAGGTAAGCTCCAATGGCTGCATGACTTC	8,850–8,881 in KF907504
YFV 8792Pr	Fam-CTGGCCAGAGAAAAGAACCCCAGACTGTGCA-BHQ1	8,792–8,822 in KF907504
JEV 8890F	TCAATAGCAACGCGGCTCTTGGA	8,890–8,912 in KF297915
JEV 9004R	TTTCCCTCTCTTCATCAACCATCTCCCAA	975–9,003 in KF297915
JEV 8935Pr	Fam-AATGGAGCACGGCGCGTGAGGCT-BHQ1	8,935–8,957 in KF297915
EnteroV_6003F	CCTGTGTAATTCCCACCACCTGTACAGA	6,754–6,781 in X80059
EnteroV_6182R	CCGTATGCAATCATCCTAAACTGGTCCAA	6,905–6,933 in X80059
EnteroV_6037Pr	FAM-CACTACTTTGAGCGGGGTGGTATGCCCTCAGG-BHQ1	6,788–6,819 in X80059
GAPDH_F	CAACAGCGACACCCACTCCT	1,046–1,065 in NM_002046.5
GAPDH_R1	CACCCTGTTGCTGTAGCCAAA	1,140–1,160 in NM_002046.5
GAPDH_prb	Fam -TGGGGCTGGCATTGCCCTCAAC-BHQ1	1,079–1,100 in NM_002046.5

For HTS, the initial and enriched cDNA libraries were indexed by PCR using Nextera-compatible oligonucleotide primers. The libraries were pooled at equal ratios. Paired-end 250-base sequencing by synthesis was performed on the MiSeq System (Illumina) using protocols provided by the manufacturer. Samples were de-multiplexed using the Illumina software, with FASTQ files generated.

### Bioinformatic analysis

Reads in the FASTQ format were filtered by quality (Q30), and the adapter sequences were removed by Trimmomatic^25^. The Bowtie 2^26^ tool was used for aligning sequencing reads to references. Alignments were processed using SAMtools^27^ and pysamstats software, and figures were generated in the R environment.

## Results

First, enrichment efficiency was estimated by comparison of the host:viral cDNA ratio in libraries before and after hybridization (Table 2).

**Table 2:** Enrichment of viral targets assessed by quantitative real-time PCR detection.

Sample	PCR assay	Ct before hybridization	dCt1 (Ct[virus]- Ct[human])	Ct after hybridization	dCt2 (Ct[virus]- Ct[human])	ddCt (dCt1- dCt2)
Human/YFV	Human	21.3	6.2	21.8	−5.1	11.3
	YFV	27.5		16.7		
Human/JEV	Human	22.0	5.0	22.3	−7.5	12.5
	JEV	27.0		14.8		
Human/E11	Human	21.4	9.3	21.2	2.3	7.0
	E11	30.7		23.5		

In unprocessed samples, delta Ct, indicating the host:pathogen ratio, was 6.2, 5.0 and 9.3 for human/YFV, human/JEV and human/E11 libraries, respectively. As a result of hybridization, this ratio changed to −5.1, −7.5 and 2.3 for human/YFV, human/JEV and human/E11 libraries, respectively. According to qPCR data, the ratio of target sequences to host sequences increased by approximately 2^11, or 2,000 times, in the case of the human/YFV library, approximately 2^12, or 4,000 times, in the case of the human/JEV library after hybridization with three pan-flavivirus oligonucleotides, and about 2^7, or 100 times, for the human/E11 library after hybridization with the FMDV-specific probe. Due to the use of the semi-quantitative PCR, the enrichment efficiency estimates should be considered as approximate; however, they at least indicate the order of magnitude of enrichment efficiency.

HTS was also performed to validate the hybridization-induced change in the host:virus cDNA ratio. Significant enrichment of target viral sequences was observed in samples after hybridization (Table 3). The number of reads mapped to viral genomes increased from single accidental reads to a significant fraction of the total. The increase in the number of viral reads corresponded well to the enrichment ratio suggested by qPCR (Table 2).

**Table 3:** Enrichment of viral targets assessed by HTS read counts.

Sample	Organism	Share of reads in HTS data, % and reads number (before hybridization)	Share of reads in HTS data, % and reads number (after hybridization)
Human/YFV	Human	92.47 (659,449/713,161)	84.43 (548,426/649,532)
	YFV	0.00 (1/713,161)	0.2 (1,315/649,532)
Human/JEV	Human	92.12 (473,999/514,540)	84.09 (631,867/751,393)
	JEV	0.00 (38/514,540)	3.37 (25,364/751,393)
Human/E11	Human	91.96 (377,820/410,873)	79.72 (197,183/247,357)
	E11	0.07 (303/410,873)	9.65 (23,880/247,357)

**Figure 1:**
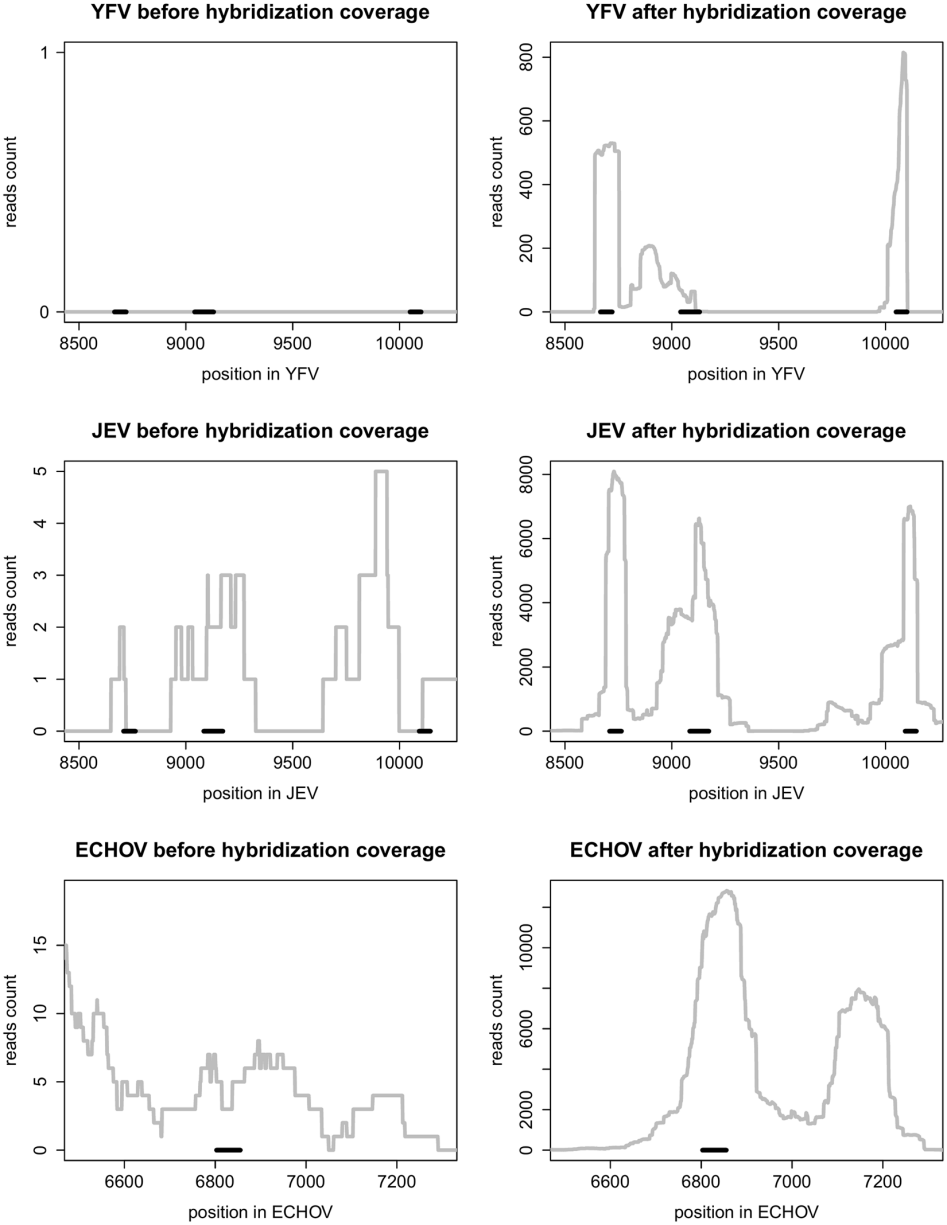
YFV, JEV, ECHOV genome fragment coverage (gray) before and after hybridization. Genome fragments, which are complementary to the biotinylated oligonucleotides, are indicated by black bars. Positions are indicated relatively to Genbank entries KF907504, KF297915, X80059, respectively.

The pattern of HTS reads coverage along the YFV, JEV and ECHOV genomes corresponded to an expected enrichment in the genome regions, which were complementary to the biotinylated oligonucleotides (Figs 1, S1). Interestingly, the pattern of reads coverage in the E11 library shows a bimodal distribution. After omitting reads shorter than 200 nucleotides from the analysis (Fig. S2), only one peak close to the probe binding site was left. Therefore, the second sequence density peak in Fig. 1 likely was due to short reads from longer cDNA library fragments that were hybridized to the oligonucleotide.

## Discussion

Rapidly evolving methods in molecular diagnostics face limitations associated with extremely high genetic diversity of viruses. Furthermore, most of the global virome remains undiscovered, resulting in a lack of reference sequences for novel pathogen identification. One way to overcome this situation is by developing a system, which allows specific enrichment of unknown virus content in processed biological material based on known homologous sequences.

We were able to increase viral cDNA content for two distinct flaviviruses (YFV and JEV) by hybridization of HTS-ready libraries with pan-flavivirus oligonucleotides. Comparison of the genomes of these viruses (KF907504 vs KF297915, respectively) using BLASTn shows a 66% identity level at 27% of the genome coverage. Oligonucleotide probes were designed based on the most conserved genome fragments and had 96.4–100.0% sequence identity (considering degenerate positions as matches) with the viruses (54/56 positions in Flavi-8469 probe vs. YFV; 88/90 in Flavi-8844 vs. YFV; 53/53 in Flavi-9834 vs. YFV; 56/56 in Flavi-8469 vs. JEV; 89/90 in Flavi-8844 vs. JEV; 52/53 in Flavi-9834 vs. JEV). The hybridization oligonucleotides showed no preference towards YFV and JEV in the enrichment experiment. The data set for the oligonucleotide design included over 100 highly diversified flavivirus sequences, therefore, comparable enrichment efficiency may be expected for any known flavivirus. To further test the specificity range of the method, we tested whether it was possible to enrich the cDNA of one virus genus with hybridization probes designed for another genus of the same family. Indeed, it was possible to enrich the content of enterovirus cDNA using an oligonucleotide designed to to the FMDV virus (79.2% sequence identity between the FMDV oligonucleotide and the enterovirus). The *Enterovirus* and *Aphthovirus* genera are very distantly related within the *Picornaviridae* family. Therefore, the specificity breadth of the method goes beyond a genus and could cover distantly related genera within a highly diversified virus family.

Importantly, cDNA enrichment by hybridization lacks theoretical specificity limitations typical of PCR, such as the strict requirement of the precise match of the three 3′-end nucleotides of the PCR primer. Any small number of additional mismatches in any part of the hybridization oligonucleotide will only reduce hybridization efficiency, but not completely prevent it. Therefore, this approach is theoretically more robust in terms of sequence variation than genus-specific PCR with degenerate primers. Moreover, the degeneracy of the probes (the number of possible unique oligonucleotides within a degenerate oligonucleotide preparation) was between 2^12^ and 2^46^, thereby greatly exceeding the maximum degeneracy of 2^8^, which is acceptable in a PCR primer.

Enrichment of cDNA libraries by hybridization involves four steps of NA copying and amplification: reverse transcription, second-strand synthesis and two rounds of library amplification before and after hybridization. Even a standard library preparation procedure results in the uneven amplification of genome fragments^28^. As expected, this bias was further deepened in our protocol. Therefore, it is advisable to use several hybridization oligonucleotides per taxon. On the other hand, using specifically designed degenerate probes, which are complementary to conserved genome regions, is much more affordable than synthesizing multiple specific oligonucleotides to the whole genome, as suggested earlier^18^.

At the present time, molecular diagnostic tools mostly rely on the specific amplification of marker sequences. In other words, while a standard molecular test answers the question about whether pathogen X is present in the sample, this does help investigators to answer the more important question, “which pathogen is present in the sample?”^29^. Moreover, the correctness of assumption about a pathogen’s presence in a sample completely depends on the qualification of a medical specialist.

To date, many tools for unbiased HTS-based pathogen detection exist. However, due to the extremely low fraction of a pathogen’s genetic material in clinical samples, its usage is only cost-effective in very special cases. The proposed technique can enrich the share of pathogen genome fragments in a library, even if the NA sequence of a pathogen is currently unknown. It can also significantly enhance the utility of HTS for diagnostics.

## Electronic supplementary material

Supplementary information
