# Supplementary material for: Enrichment of Viral Nucleic Acids by Solution Hybrid Selection with Genus Specific Oligonucleotides
Source: Sci Rep. 2017 Aug 29;7:9752. doi: 10.1038/s41598-017-10342-w (PMC5575070; doi:10.1038/s41598-017-10342-w)
Supplement: Supplementary file 1 — Supplementary information [file 41598_2017_10342_MOESM1_ESM.pdf]

Enrichment of Viral Nucleic Acids by Solution Hybrid Selection with Genus Specific Oligonucleotides

Deviatkin AA[1,2]*, Lukashev AN[1,3] , Markelov ML[2], Gmyl LV[1], Shipulin GA[4]

1. Chumakov Federal Scientific Center for Research and Development of Immune and Biological Products of Russian Academy of Sciences,

2. Research Institute of Occupational Health, Moscow, Russian Federation,

3. Institute of Molecular Medicine, Sechenov First Moscow State Medical University, Moscow, Russia,

4. Federal Budget Institute of Science Central Research Institute for Epidemiology, Moscow, Russian Federation.

* Corresponding author

E-mail: andreideviatkin@gmail.com (AAD)

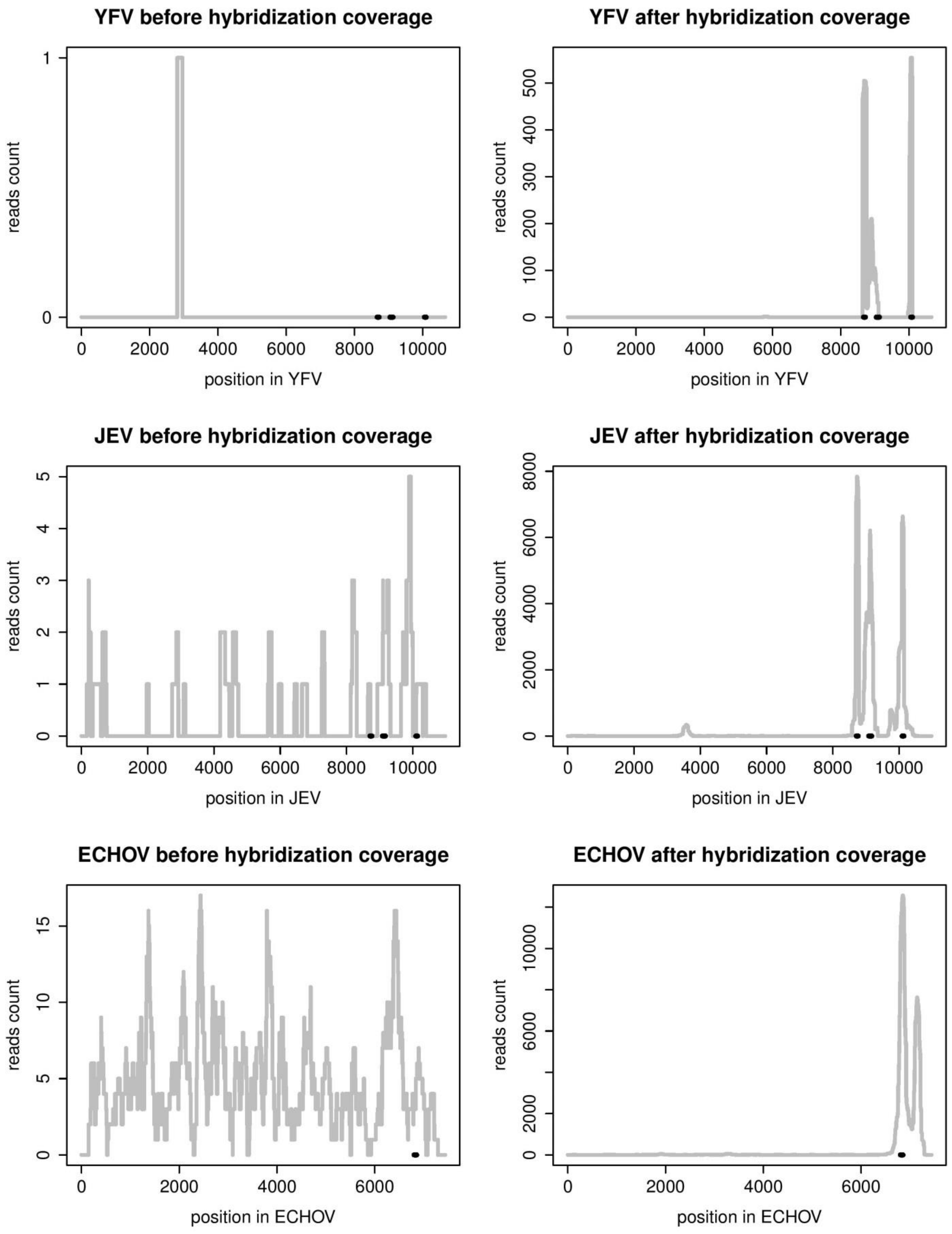

Supplementary Figure S1. YFV, JEV, E11 genome coverage before and after hybridization. Genome fragments complementary to biotinylated oligonucleotides are indicated by the black bar. Genome positions are given according to GenBank records KF907504, KF297915, X80059.

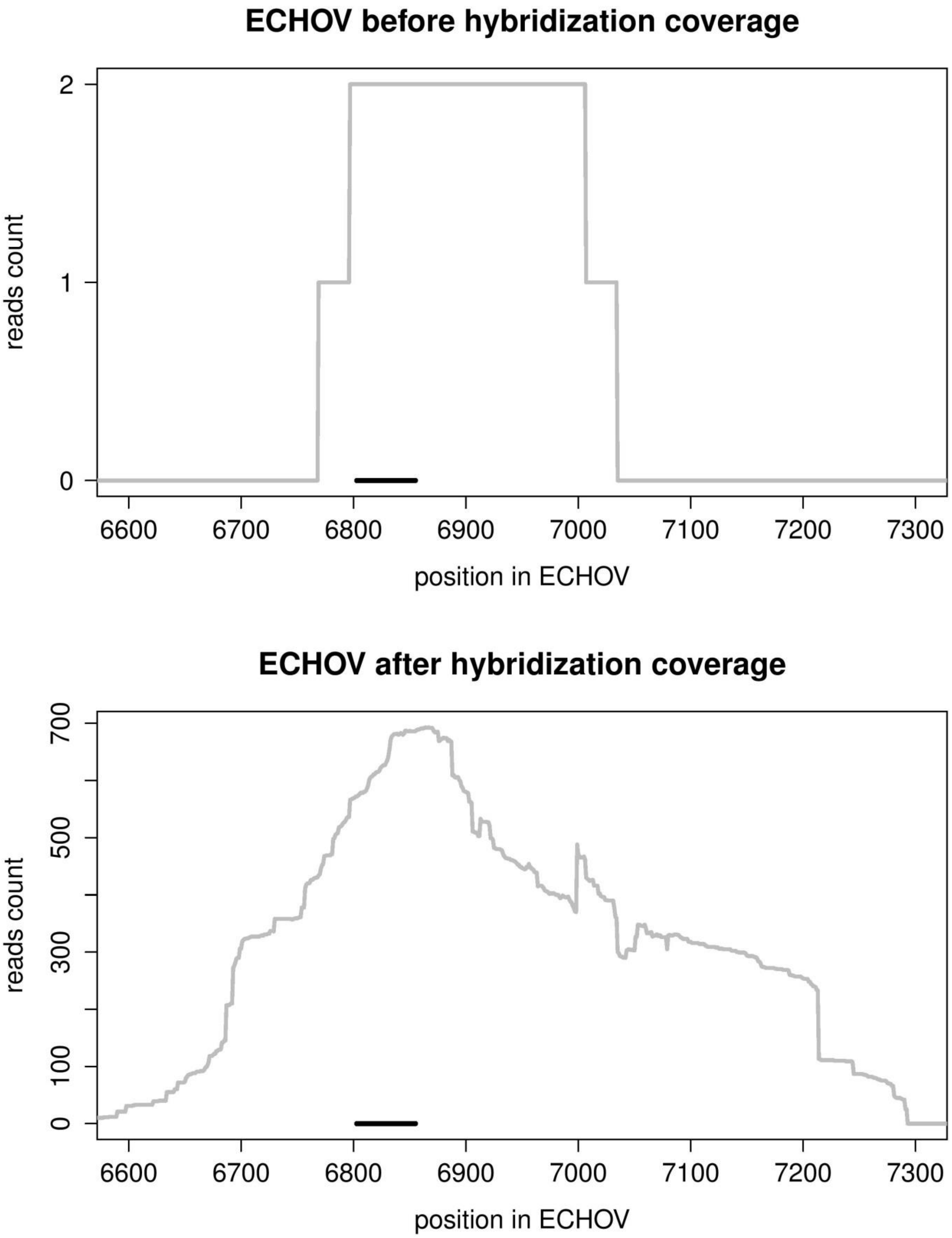

Supplementary Figure S2. E11 genome coverage before and after hybridization (reads shorter than 200 nt were filtered out of the analysis). Genome fragments complementary to biotinylated oligonucleotide (FMDV) is indicated by the black bar. Positions are indicated according to GenBank record X80059.
